# Necroptosis‐related lncRNA to establish novel prognostic signature and predict the immunotherapy response in breast cancer

**DOI:** 10.1002/jcla.24302

**Published:** 2022-03-01

**Authors:** Fangyue Chen, Jun Yang, Min Fang, Yanmei Wu, Dongwei Su, Yuan Sheng

**Affiliations:** ^1^ Department of General Surgery Changhai Hospital Navy Military Medical University Shanghai China; ^2^ Department of General Surgery 63650 Military Hospital China

**Keywords:** breast cancer, lncRNA, necroptosis, TIDE, TMB

## Abstract

**Background:**

Necroptosis is a type of programmed cell death, and recent researches have showed that lncRNAs could regulate the process of necroptosis in multiple cancers. We tried to screen necroptosis‐related lncRNAs and investigate the immune landscape in breast cancer (BC).

**Methods:**

The samples of breast normal and cancer tissue were acquired from TCGA and GTEx databases. A risk prognostic model was constructed based on the identified necroptosis‐related lncRNAs by Cox regression and least absolute shrinkage and selection operator (LASSO) method. Moreover, the forecast performance of this model was verified and accredited by synthetic approach. Subsequently, an accurate nomogram was constructed to predict the prognosis of BC patients. The biological differences were investigated through GO, GSEA, and immune analysis. The immunotherapy response was estimated through tumor mutation burden (TMB) and tumor immune dysfunction and exclusion (TIDE) score.

**Results:**

A total of 251 necroptosis‐related lncRNAs were identified by differential coexpression analysis, and SH3BP5‐AS1, AC012073.1, AC120114.1, LINC00377, AL133467.1, AC036108.3, and AC020663.2 were involved in the risk model, which had an excellent concordance with the prediction. The pathway analyses showed that immune‐related pathways were relevant to the necroptosis‐related lncRNAs risk model. And the risk score was significantly correlated with immune cell infiltration, as well as the ESTIMATE score. Most notably, the patients of higher risk score were characterized with increased TMB and decreased TIDE score, indicating that these patients showed better immune checkpoint blockade response.

**Conclusion:**

These findings were conducive to understand the function of necroptosis‐related lncRNAs in BC and provide a potential promising therapeutic strategy for BC.

## Graphical abstract

Seven necroptosis‐related lncRNAs, SH3BP5‐AS1, AC012073.1, AC120114.1, LINC00377, AL133467.1, AC036108.3, and AC020663.2 were involved in the risk model. In addition, the forecast performance of this model was verified and accredited. Most notably, the patients of higher risk score were characterized with increased TMB and decreased TIDE score, indicating that these patients showed better immune checkpoint blockade response.

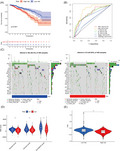

## 1 INTRODUCTION

Worldwide, breast cancer (BC) is one of the most prevalent types of malignancy and a major cause of cancer death.^1^ The treatment landscape of patients with breast cancer has been rapidly evolving in recent years, and optimal therapy paradigm for breast cancer depends on subpopulations of patients.

Necroptosis, mainly mediated by receptor‐interacting protein kinase 1 (RIPK1), RIPK3, and mixed lineage kinase domain like pseudokinase (MLKL), belongs to the category of programmed cell death.^2^, ^3^, ^4^

Increasing evidence demonstrated that necroptosis played a pivotal role in the occurrence and progression of multifarious diseases, such as neurodegenerative diseases, ischemic cardiovascular, and cancer metastasis.^5^ Besides, necroptosis had dual impact on promoting and suppressing tumor growth in multiple tumor types.^6^, ^7^, ^8^ Accordingly, regulating tumor necroptosis may be a novel and potential therapeutic strategy for BC.^9^

Long noncoding RNAs (lncRNAs) have attracted growing focus as tumor markers for early‐stage detection, diagnosis, prognosis, and prediction of drug therapy response.^10^, ^11^ Different lncRNAs regulate the expression of genes through epigenetic regulation or transcriptional alternation, and the aberrant expression of lncRNAs is closely linked to tumorigenesis, metastasis, and chemoresistance of cancer.^12^, ^13^, ^14^ Up to date, it has been reported that some lncRNAs engaged in modulating the process of necroptosis by interacting with miRNA to regulate necroptosis‐related genes products.^15^, ^16^, ^17^ Given that, further insight into the function of necroptosis‐related lncRNAs in BC may provide novel approach for precise treatment and individualized management.

## 2 MATERIALS AND METHODS

### 2.1 Normal and tumor sample extraction from dataset

The transcriptome RNA‐seq datasets (HTSeq‐Counts and FPKM) of female breast cancer (BC) and normal samples were acquired from The Cancer Genome Atlas (TCGA) and Genotype‐Tissue Expression Project (GTEx), respectively. The HTSeq‐Counts value matrix was used to search for the differentially expressed (DE) lncRNAs, while the FPKM values were transformed to TPM values for other analyses. After excluding the sample of male, 191 normal tissue samples (79 samples from GTEx dataset) and 1086 BC samples were obtained from two datasets. After ruling out the missing overall survival time, 1057 cases with survival time and 908 cases with full clinical pathology information were extracted for following analyses.

### 2.2 Identification of Necroptosis‐related lncRNA

According to necroptosis gene set M24779.gmt and previous literature search, 67 necroptosis‐related genes were collected for next identification.^18^ Then, we used GENCODE annotation file to identify 14,106 lncRNAs in the TCGA combined with GTEx datasets (http://cancergenome.nih.gov/abouttcga, http://www.gtexportal.org). The DE lncRNAs were screened by DESeq2 R package with the standard of |log2 fold change (FC) | >1, false discovery rate (FDR) <0.05, and *p* adjusted <0.05. The Pearson correlation algorithm was used to identify necroptosis‐related DE lncRNA with correlation filter >0.4 and *p* < 0.001. After the completion of screening steps, 251 necroptosis‐related lncRNAs were retrieved for further analysis. The analyses were based on limma R package.

### 2.3 Establishment and validation of prognostic risk assessment model

Preliminarily, the prognostic‐classified lncRNAs were selected by using univariate Cox (uni‐Cox) regression with *p* value < 0.05. Then, LASSO regression analysis was made to filter necroptosis‐related lncRNA with 10‐fold cross‐validation. Further, the necroptosis‐related lncRNAs screened by LASSO method were used for multivariate (multi‐Cox) proportional hazards regression and risk model construction. The risk score was calculated by using following formula: risk score = $\Sigma_{k=1}^{n}$ expression (lncRNA^k^) × coefficient(lncRNA^k^). The median risk score that calculated by the above formula was used to stratify the BC patients into low‐ and high‐risk groups. The chi‐square test was used to validate the correlation of the clinical features and the risk group. The independent variables were assessed by uni‐Cox and multi‐Cox regression analyses, respectively. Receiver operating characteristic (ROC) curves and concordance index(C‐index) were subsequently applied to measure the precision of the model. The analyses were based on survival, caret, glmnet, rms, survminer, and timeROC R packages.

### 2.4 Predictive nomogram construction and calibration

With rms R package, the nomogram was set up based on risk group, age, and clinicopathological factors. The nomogram aimed to evaluating the predictive efficacy of risk score we got for 1‐, 3‐, 5‐, and 10‐year overall survival rates. Subsequently, the calibration curve was developed to illustrate the prediction power of the established nomogram model.

### 2.5 PCA, GO, and GSEA analysis

The principal component analysis (PCA) was used to classify BC samples through necroptosis‐related lncRNAs expression patterns. Additionally, the spatial distribution of samples was displayed through three‐dimensional scatterplot. We identified the differential genes among the low‐ and high‐risk groups for subsequent Gene Ontology (GO) analysis, aiming to investigate the relevant biological process. Furtherly, differentially expressed KEGG pathways in two groups were identified by Gene Set Enrichment Analysis (GSEA). The KEGG gene set(c2.cp.kegg.v7.0.symbols.gmt) was derived from the website (https://www.gsea‐msigdb.org/). The limma, org. Hs.eg.db, clusterProfiler, and enrichplot package based on R 4.1.1 were used for the analysis. The threshold of significantly enriched biological processes and pathways was set as *p* < 0.05 and FDR <0.25.

### 2.6 Tumor microenvironment in low‐ and high‐risk groups

The correlations between risk score and tumor‐infiltrating immune cells (TIICs) were evaluated by CIBERSORT algorithm. Furthermore, with ESTIMATE R package,^19^ we calculated the tumor microenvironment (TME) score, including stromal score, immune score, and estimate score between low‐ and high‐risk groups.

### 2.7 Tumor mutation burden and Tumor Immune Dysfunction and Exclusion score

The somatic mutation file (TCGA.BRCA.varscan.DR‐10.0.somatic) was obtained from the TCGA website. The original mutation annotation format (MAF) was divided into two groups according to the risk score. Then, we calculated the tumor mutation burden (TMB) score according to the somatic mutation data for each patient in the two groups. The foregoing analyses were based on maftools R package. Potential immune checkpoint blockade (ICB) response was assessed by tumor immune dysfunction and exclusion (TIDE) algorithm.^20^ Finally, we used pRRophetic R package to calculate the semi‐inhibitory concentration (IC50) values of chemotherapeutic drugs.

## 3 RESULTS

### 3.1 Altered Expression of the necroptosis‐related lncRNA in BRCA

We analyzed the necroptosis‐related lncRNA expression level in 1,086 BC samples and 191 normal samples from the TCGA and GTEx datasets, and 2,848 DE lncRNAs were obtained. Then, 251 necroptosis‐related lncRNAs were identified in the DE lncRNAs by the Pearson correlation algorithm.

### 3.2 Construction and verification of prognosis risk assessment model

**FIGURE 1:**
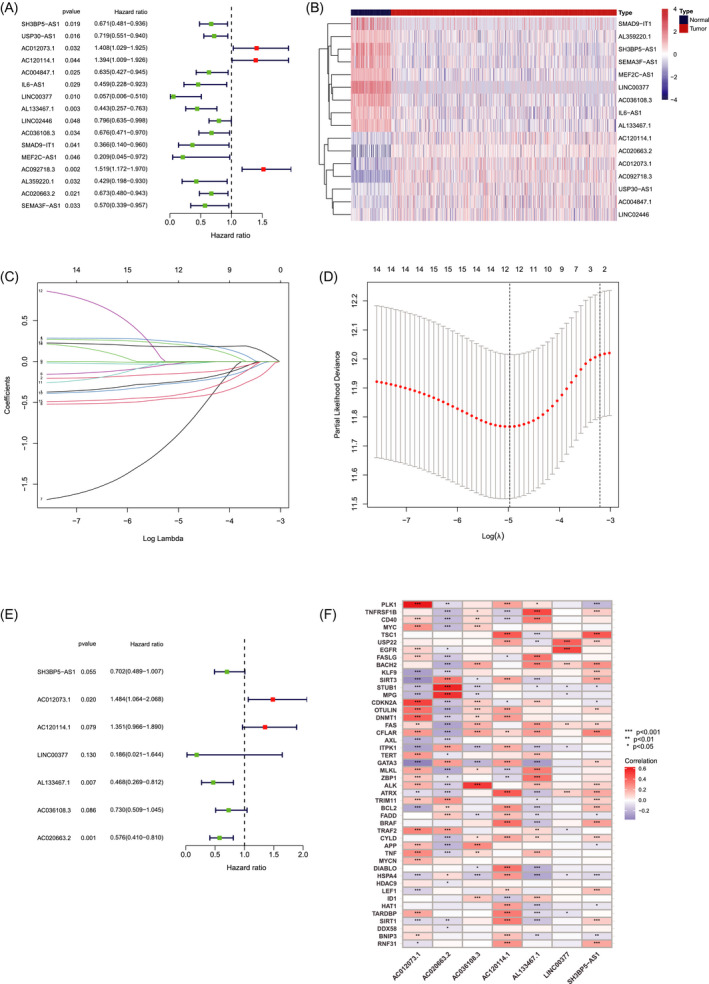
Identification of prognostic necroptosis‐related lncRNAs in BC. (A) The prognostic lncRNAs extracted by uni‐Cox regression analysis. (B) The heatmap of 16 lncRNAs expression. (C) The 10‐fold cross‐validation for tuning parameter selection in the LASSO model. (D) The LASSO coefficient profile of 16 survival‐associated lncRNAs. (E) 7 lncRNAs identified by multi‐Cox proportional hazard model. (F) Correlations between lncRNAs in the risk model and necroptosis‐related genes

**FIGURE 2:**
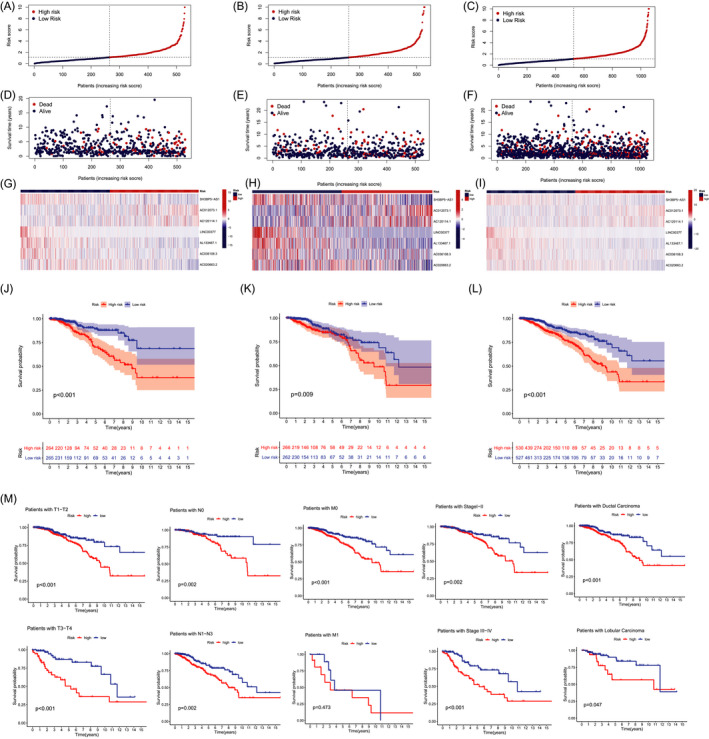
Prognosis of the risk model in the different sets. (A–C) Demonstration of risk model of the training, validation, and complete sets. (D–F) Survival time and clinical endpoint in the training, validation, and complete sets. (G–I) The heatmap of 7 lncRNAs expression in the training, validation, and complete sets. (J–L) K‐M survival curves of OS of patients between the two groups in the training, validation, and complete sets. (M) K‐M survival curves of OS prognostic value stratified by clinicopathologic characteristics in the complete set

**FIGURE 3:**
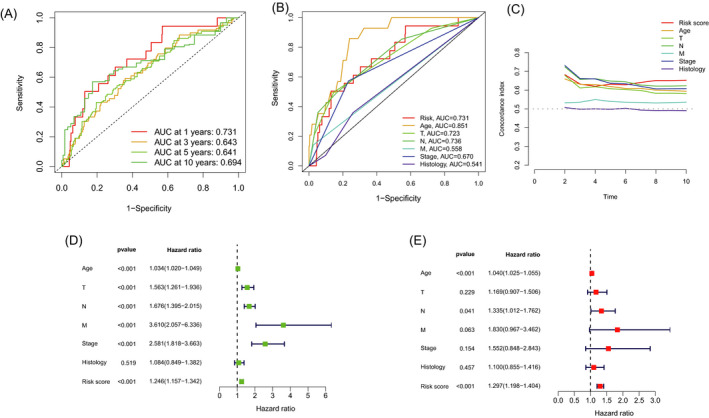
Verification of prognosis risk assessment model. (A) The 1‐, 3‐, 5‐, and 10‐year ROC curves of the complete sets. (B) The ROC curves of risk score and clinicopathologic features. (C) The C‐index curves of risk model. (D) Uni‐Cox analyses of clinicopathologic factors and risk score with OS. (E) Multi‐Cox analyses of clinicopathologic factors and risk score with OS

Firstly, 16 lncRNAs was extracted by means of the uni‐Cox regression analysis. Then, 12 lncRNAs were acquired by LASSO analysis, and 7 of which were brought in the multi‐Cox proportional hazards model (Figure 1A‐E). Finally, we got the risk score with the formula from multivariate Cox regression: Risk score = SH3BP5‐AS1 × (−0.3537) + AC012073.1 × (0.3945) + AC120114.1 × (0.3010) + LINC00377 × (−1.6837) + AL133467.1 × (−0.7597) + AC036108.3 × (−0.3151) + AC020663.2 × (−0.5513). In the complete set, training and validation partitions, all patients in the high‐risk group had a significantly shorter overall survival duration (Figure 2A‐I). The same results were displayed in the different clinicopathologic characteristics (Figure 2M). The area under the 1‐,3‐,5‐, and 10‐year ROC curve (AUC) was 0.731, 0.643, 0.641, and 0.694, respectively (Figure 3A). At the 10‐year ROC of the model, the AUC of risk score was 0.731, demonstrating strong predictive ability compared with other clinicopathology features (Figure 3B). The 1‐year C‐index in the risk model was 0.726 (Figure 3C). In uni‐Cox and multi‐Cox regression, the hazard ratio (HR) of the risk score were 1.246 and 1.279, respectively (both *p* value < 0.001) (Figure 3D‐E).

### 3.3 Construction of nomogram

**FIGURE 4:**
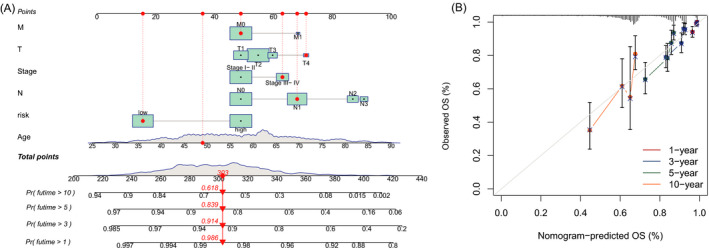
Nomogram and calibration curves of the model. (A) Nomogram for predicting overall survival. (B) The calibration curves for 1‐, 3‐, 5‐, and 10‐year OS

Based on risk score, age, and clinicopathological factors, a nomogram was developed for predicting the 1‐, 3‐, 5‐, and 10‐year OS incidences (Figure 4A). The calibration plots were applied to testify that whether the nomogram had an excellent concordance with the prediction (Figure 4B), which exhibited the good consistency with the actual observation.

### 3.4 The PCA and biological pathways analyses

**FIGURE 5:**
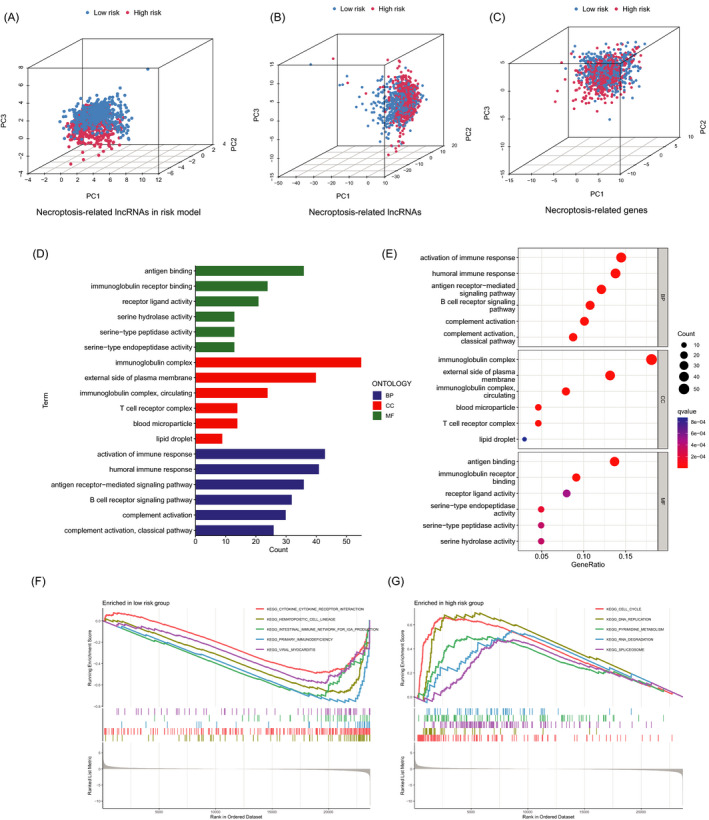
The PCA and functional analyses of patients in two groups. (A‐C) The PCA 3D scatterplot of sample distribution based on necroptosis‐related lncRNAs in risk model, necroptosis‐related lncRNAs, and necroptosis‐related genes, respectively. (D‐E) The GO analysis. (F‐G) The GSEA analysis

The three‐dimensional scatter diagram of the PCA respectively showed the distribution of different patterns. The samples grouped by risk score had distinct aggregation feature (Figure 5A‐C). The results of Gene Ontology (GO) analysis include positive regulation of activation of immune response, humoral immune response, and B‐cell receptor signaling pathway (Figure 5D‐E). The results from the GSEA analysis showed different biological functions between the low‐ and high‐risk groups, such as cell cycle, DNA replication, pyrimidine metabolism, RNA degradation, spliceosome, and immune network (Figure 5F‐G). Therefore, we tried to make an immune‐related analysis based on the risk model.

### 3.5 Investigation of immune signature in risk groups

**FIGURE 6:**
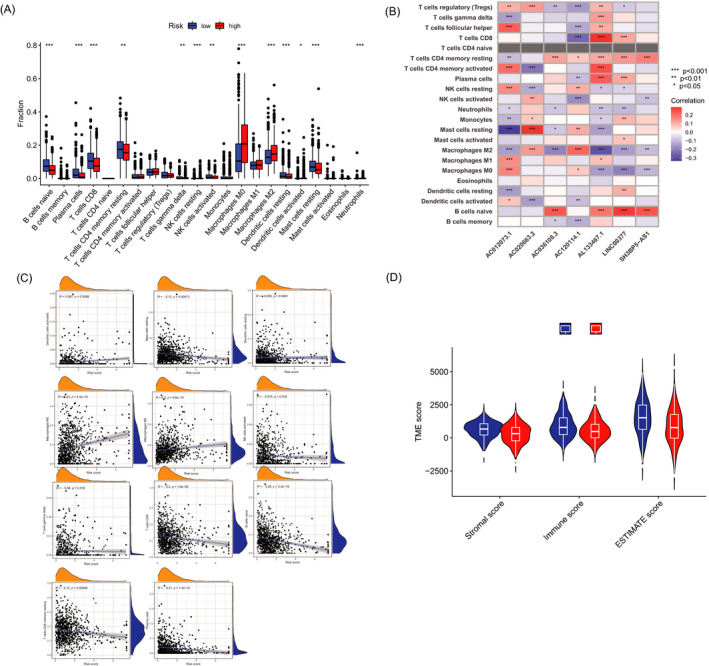
Immune signature in two groups. (A) Expression of immune cells in the low‐ and high‐risk groups. (B) Correlations between the TIICs and 7 necroptosis‐related lncRNAs in the proposed model. (C) Correlations between risk score and immune cell types. (D) TME score in the in two groups

Significant differences in the immune cell infiltration were observed between the two groups (Figure 6A), and the intricate correlations existed between TIICs and 7 necroptosis‐related lncRNAs (Figure 6B). As shown in the scatter diagrams, dendritic cells activated, M0 macrophages, and M2 macrophages were positively correlated with the aforesaid risk scores, by contrast, the other TIICs had negative correlations. (Figure 6C). As for the TME score, high‐risk patients showed lower stromal scores, immune scores, and ESTIMATE score than low‐risk patients (Figure 6D).

### 3.6 TMB, TIDE, and therapeutic drug sensitivity

**FIGURE 7:**
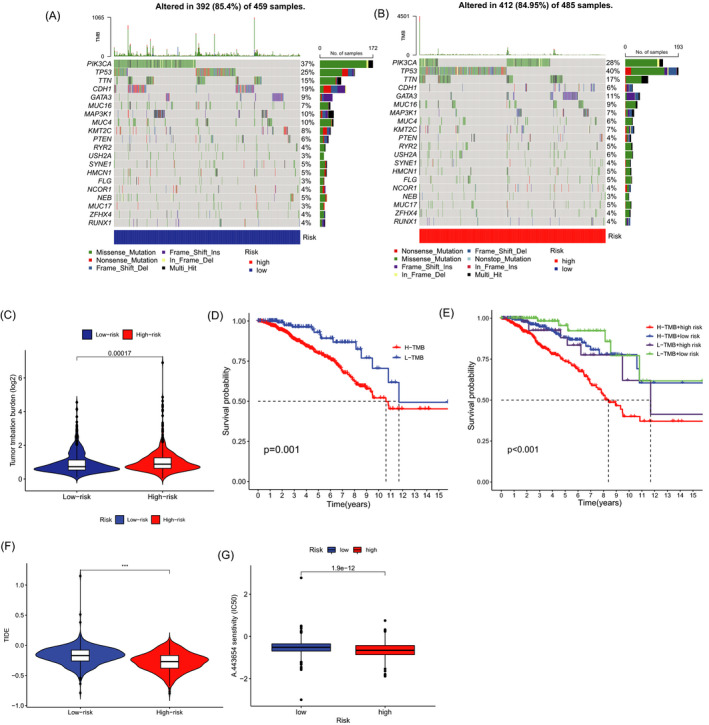
TMB, TIDE, and Chemotherapeutic Sensitivity. (A‐B) The waterfall plot of somatic mutation features in two groups. (C) TMB between low‐ and high‐risk groups. (D) K–M survival curves between the high‐TMB and low‐TMB groups. (E) K‐M survival curves between the two groups. (F) TIDE score between two groups. (G) IC50 difference in A.443654

Then, we analyzed the variations of the somatic mutations in two risk groups. The ten highest mutated genes were PIK3CA, TP53, TTN, CDH1, GATA3, MUC16, MAP3K1, MUC4, KMT2C, and PTEN. Patients in high‐risk group had markedly higher frequencies of TP53 mutation, and the opposite result was discovered with the alternation of PIK3CA and CDH1(Figure 7A–B). Compared with low‐risk group, patients in high‐risk group had higher TMB (Figure 7C). Besides, patients in the high‐risk and high‐TMB group had worst prognosis compared with the other group (Figure 7D‐E). The TIDE score was significantly lower in high‐risk group compared with low‐risk group (Figure 7F). Through drug sensitivity comparison, we found that A.443654, an AKT inhibitor, showed different IC50 between two groups, and BC patients in high‐risk group were more sensitive to this drug (Figure 7G).

## 4 DISCUSSION

Necroptosis involved in immune responses and tumor microenvironment, and the benefits of activation of necroptosis pathways combined with immune checkpoint blockade have been demonstrated in recent study.^21^ Nowadays, it has been proved that lncRNAs engage in cancer‐related cellular pathways and have good predictive power in prognosis and diagnosis.^22^, ^23^ Emerging studies have tried to establish novel and effective lncRNAs pattern risk models in malignancy,^24^, ^25^ as well as discover molecular character and potential therapy targets of breast cancer patients.^26^, ^27^ Until now, the patterns of necroptosis‐related lncRNAs in BC and the potential capability of predicting the prognosis has not been elucidated.

In this study, we established a risk model with 7 necroptosis‐related lncRNAs, including SH3BP5‐AS1, AC012073.1, AC120114.1, LINC00377, AL133467.1, AC036108.3, and AC020663.2. Then, the patients were divided into low‐ and high‐risk groups according to the median values. The ROC and C‐index curve fatherly validated the prognostic precision of the risk score. We found that the risk score could be a yardstick for predicting prognosis. Subsequently, a nomogram was constructed for predicting prognosis of BC patients, which had an excellent concordance with the prediction.

The 3D scatterplot of the PCA showed that patients categorized by necroptosis‐related lncRNAs exhibited distinct inherent biological feature. The results of Gene Ontology (GO) analysis demonstrated that activation of immune response, humoral immune response, and B‐cell receptor signaling pathway played an important role in biological pathways. Besides, the results of GSEA showed different enrichment of genes in KEGG, including cell cycle, DNA replication, pyrimidine metabolism, RNA degradation, spliceosome, and immune network. Although breast cancer is not regarded as a highly immunogenic cancer in the past, but tumor immune microenvironment impacts on a subset of breast cancers and partial patients might be suitable to immune checkpoint blockade treatment strategies after evaluation.^28^, ^29^ Based on the above finding, we tried to make an immunity analysis by CIBERSORT and ESTIMATE method in the risk model. The risk score was positively correlated with dendritic cells activated, M0 macrophages, and M2 macrophages. ESTIMATE is a method to assess the immune cells infiltration and tumor microenvironment according to gene expression. In this study, immune scores, stromal scores, and estimate scores of high‐risk groups were significantly lower.

The somatic mutation analysis showed that the samples in high‐risk group had more frequent TP53 mutation mutated and TP53 mutations may boost immunotherapy activity in BC according to previous study.^30^ Although immunotherapy has been applied successfully in some tumor types, not all the breast cancer patients can benefit from this treatment.^31^ Therefore, it is indispensable to select appropriate biomarkers to decern the patients who are more sensitive to immunotherapy.

Hypermutated breast cancer patients may benefit from PD‐1 inhibitors,^32^ and high tumor mutation burden (TMB) is related to better therapeutic effect of immune checkpoint blockade (ICB).^33^, ^34^ In this study, the patients in the high‐risk group showed a higher TMB. Tumor immune dysfunction and exclusion (TIDE) algorithm is a method for predicting ICB response in cancer.^20^ A higher TIDE score is associated with worse ICB response and has high accuracy in predicting the survival outcome of cancer patients treated with ICB.^35^ Some recent research supported its application in predicting the therapeutic effect of ICB.^36^, ^37^ In our study, the TIDE score was significantly lower in high‐risk group. In conclusion, based on the evaluation of TMB and TIDE score, the patients in high‐risk group showed better ICB response. In addition, we used pRRophetic R package to calculate the IC50 values of chemotherapeutic drugs, and the patients with high‐risk score were more sensitive to A‐443654.^38^

There are still limitation and weaknesses in our study. Firstly, the analysis results were not validated in vitro and in vivo, and the biological function needs to be furtherly elucidated. Secondly, in view of complexity, we did not clarify the relationship between necroptosis‐related lncRNA and tumor‐infiltrating immune cells. Thirdly, in the retrospective study, there may be some biases in the case inclusion and data processing. The collection of clinical samples and external validation will be implemented in the future.

## 5 CONCLUSION

A well‐validated risk model was constructed based on 7 necroptosis‐related lncRNAs, including SH3BP5‐AS1, AC012073.1, AC120114.1, LINC00377, AL133467.1, AC036108.3, and AC020663.2. The BC patients in the high‐risk group had worse clinical outcomes. Besides, the high‐risk patients demonstrated higher TMB and lower TIDE score, indicating the better immune checkpoint blockade response. These findings pointed novel ways of BC prognosis estimation and optimal treatment strategy.

## Data Availability

The data that support the findings of this study are openly available in TCGA and GTEx at http://cancergenome.nih.gov/abouttcga and http://www.gtexportal.org, respectively.

## References

[1] Bray F , Ferlay J , Soerjomataram I , Siegel RL , Torre LA , Jemal A . Global cancer statistics. CA Cancer J Clinic. 2018;68(6):394‐424. 10.3322/caac.21492 30207593

[2] Rodriguez DA , Weinlich R , Brown S , et al. Characterization of RIPK3‐mediated phosphorylation of the activation loop of MLKL during necroptosis. Cell Death Differ. 2016;23(1):76‐88. 26024392 10.1038/cdd.2015.70 PMC4815980

[3] Moujalled DM , Cook WD , Murphy JM , Vaux DL . Necroptosis induced by RIPK3 requires MLKL but not Drp1. Cell Death Dis. 2014;5:e1086. 24577084 10.1038/cddis.2014.18 PMC3944236

[4] Grootjans S , Vanden Berghe T , Vandenabeele P . Initiation and execution mechanisms of necroptosis: an overview. Cell Death Differ. 2017;24(7):1184‐1195. 28498367 10.1038/cdd.2017.65 PMC5520172

[5] Gong Y , Fan Z , Luo G , et al. The role of necroptosis in cancer biology and therapy. Mol Cancer. 2019;18(1):100. 31122251 10.1186/s12943-019-1029-8 PMC6532150

[6] Strilic B , Yang L , Albarrán‐Juárez J , et al. Tumour‐cell‐induced endothelial cell necroptosis via death receptor 6 promotes metastasis. Nature. 2016;536(7615):215‐218. 27487218 10.1038/nature19076

[7] Seifert L , Werba G , Tiwari S , et al. The necrosome promotes pancreatic oncogenesis via CXCL1 and mincle‐induced immune suppression. Nature. 2016;532(7598):245‐249. 27049944 10.1038/nature17403 PMC4833566

[8] Qin X , Ma D , Tan YX , Wang HY , Cai Z . The role of necroptosis in cancer: a double‐edged sword? Biochim Biophys Acta Rev Cancer. 2019;1871(2):259‐266. 30716362 10.1016/j.bbcan.2019.01.006

[9] Shen F , Pan X , Li M , Chen Y , Jiang Y , He J . Pharmacological inhibition of necroptosis promotes human breast cancer cell proliferation and metastasis. Onco Targets Ther. 2020;13:3165‐3176. 32368076 10.2147/OTT.S246899 PMC7170643

[10] Bhan A , Soleimani M , Mandal SS . Long noncoding RNA and cancer: a new paradigm. Cancer Res. 2017;77(15):3965‐3981. 28701486 10.1158/0008-5472.CAN-16-2634 PMC8330958

[11] Lv W , Hu W , Chi L , Zhang L . Identification and validation of m6A‐related lncRNA signature as potential predictive biomarkers in breast cancer. Front Oncol. 2021;11:745719. 34722303 10.3389/fonc.2021.745719 PMC8555664

[12] Prensner JR , Chinnaiyan AM . The emergence of lncRNAs in cancer biology. Cancer Discov. 2011;1(5):391‐407. 22096659 10.1158/2159-8290.CD-11-0209 PMC3215093

[13] Schmitt AM , Chang HY . Long noncoding RNAs in cancer pathways. Cancer Cell. 2016;29(4):452‐463. 27070700 10.1016/j.ccell.2016.03.010 PMC4831138

[14] Yousefi H , Maheronnaghsh M , Molaei F , et al. Long noncoding RNAs and exosomal lncRNAs: classification, and mechanisms in breast cancer metastasis and drug resistance. Oncogene. 2020;39(5):953‐974. 31601996 10.1038/s41388-019-1040-y

[15] Liu Y , Chen Q , Zhu Y , et al. Non‐coding RNAs in necroptosis, pyroptosis and ferroptosis in cancer metastasis. Cell Death Discov. 2021;7(1):210. 34381023 10.1038/s41420-021-00596-9 PMC8358062

[16] Jiang N , Zhang X , Gu X , Li X , Shang L . Progress in understanding the role of lncRNA in programmed cell death. Cell Death Discov. 2021;7(1):30. 33558499 10.1038/s41420-021-00407-1 PMC7870930

[17] Guan YX , Zhang M‐Z , Chen X‐Z , et al. Lnc RNA SNHG20 participated in proliferation, invasion, and migration of breast cancer cells via miR‐495. J Cell Biochem. 2018;119(10):7971‐7981. 29236315 10.1002/jcb.26588

[18] Zhao Z , Liu H , Zhou X , et al. Necroptosis‐related lncRNAs: predicting prognosis and the distinction between the cold and hot tumors in gastric cancer. J Oncol. 2021;2021:6718443. 34790235 10.1155/2021/6718443 PMC8592775

[19] Yoshihara K , Shahmoradgoli M , Martínez E , et al. Inferring tumour purity and stromal and immune cell admixture from expression data. Nat Commun. 2013;4:2612. 24113773 10.1038/ncomms3612 PMC3826632

[20] Jiang P , Gu S , Pan D , et al. Signatures of T cell dysfunction and exclusion predict cancer immunotherapy response. Nat Med. 2018;24(10):1550‐1558. 30127393 10.1038/s41591-018-0136-1 PMC6487502

[21] Snyder AG , Hubbard NW , Messmer MN , et al. Intratumoral activation of the necroptotic pathway components RIPK1 and RIPK3 potentiates antitumor immunity. Sci Immunol. 2019;4(36):eaaw2004. doi:10.1126/sciimmunol.aaw2004 31227597 PMC6831211

[22] Marchese FP , Raimondi I , Huarte M . The multidimensional mechanisms of long noncoding RNA function. Genome Biol. 2017;18(1):206. 29084573 10.1186/s13059-017-1348-2 PMC5663108

[23] Chi Y , Wang D , Wang J , Yu W , Yang J . Long non‐coding RNA in the pathogenesis of cancers. Cells. 2019;8(9):1015. 10.3390/cells8091015 PMC6770362 31480503

[24] Liu Z , Mi M , Li X , Zheng X , Wu G , Zhang L . A lncRNA prognostic signature associated with immune infiltration and tumour mutation burden in breast cancer. J Cell Mol Med. 2020;24(21):12444‐12456. 32967061 10.1111/jcmm.15762 PMC7687003

[25] Guo Y , Qu Z , Li D , et al. Identification of a prognostic ferroptosis‐related lncRNA signature in the tumor microenvironment of lung adenocarcinoma. Cell Death Discov. 2021;7(1):190. 34312372 10.1038/s41420-021-00576-z PMC8313561

[26] Li X , Li Y , Yu X , Jin F . Identification and validation of stemness‐related lncRNA prognostic signature for breast cancer. J Transl Med. 2020;18(1):331. 32867770 10.1186/s12967-020-02497-4 PMC7461324

[27] Lv W , Tan Y , Zhao C , et al. Identification of pyroptosis‐related lncRNAs for constructing a prognostic model and their correlation with immune infiltration in breast cancer. J Cell Mol Med. 2021;25(22):10403‐10417. 34632690 10.1111/jcmm.16969 PMC8581320

[28] Burugu S , Asleh‐Aburaya K , Nielsen TO . Immune infiltrates in the breast cancer microenvironment: detection, characterization and clinical implication. Breast Cancer. 2017;24(1):3‐15. 27138387 10.1007/s12282-016-0698-z

[29] Ruffell B , Au A , Rugo HS , Esserman LJ , Hwang ES , Coussens LM . Leukocyte composition of human breast cancer. Proc Natl Acad Sci USA. 2012;109(8):2796‐2801. 21825174 10.1073/pnas.1104303108 PMC3287000

[30] Liu Z , Jiang Z , Gao Y , Wang L , Chen C , Wang X . TP53 mutations promote immunogenic activity in breast cancer. J Oncol. 2019;2019:5952836. 31275382 10.1155/2019/5952836 PMC6582869

[31] Adams S , Gatti‐Mays ME , Kalinsky K , et al. Current landscape of immunotherapy in breast cancer: a review. JAMA Oncol. 2019;5(8):1205‐1214. 30973611 10.1001/jamaoncol.2018.7147 PMC8452050

[32] Barroso‐Sousa R , Jain E , Cohen O , et al. Prevalence and mutational determinants of high tumor mutation burden in breast cancer. Ann Oncol. 2020;31(3):387‐394. 32067680 10.1016/j.annonc.2019.11.010

[33] Chaudhary K , Poirion OB , Lu L , Garmire LX . Deep learning‐based multi‐omics integration robustly predicts survival in liver cancer. Clin Cancer Res. 2018;24(6):1248‐1259. 28982688 10.1158/1078-0432.CCR-17-0853 PMC6050171

[34] Yarchoan M , Hopkins A , Jaffee EM . Tumor mutational burden and response rate to PD‐1 inhibition. N Engl J Med. 2017;377(25):2500‐2501. 29262275 10.1056/NEJMc1713444 PMC6549688

[35] Keenan TE , Burke KP , Van Allen EM . Genomic correlates of response to immune checkpoint blockade. Nat Med. 2019;25(3):389‐402. 30842677 10.1038/s41591-019-0382-x PMC6599710

[36] Liu D , Schilling B , Liu D , et al. Integrative molecular and clinical modeling of clinical outcomes to PD1 blockade in patients with metastatic melanoma. Nat Med. 2019;25(12):1916‐1927. 31792460 10.1038/s41591-019-0654-5 PMC6898788

[37] Cao R , Yuan L , Ma B , Wang G , Tian Y . Immune‐related long non‐coding RNA signature identified prognosis and immunotherapeutic efficiency in bladder cancer (BLCA). Cancer Cell Int. 2020;20:276. 32607061 10.1186/s12935-020-01362-0 PMC7320553

[38] Han EK , Leverson JD , McGonigal T , et al. Akt inhibitor A‐443654 induces rapid Akt Ser‐473 phosphorylation independent of mTORC1 inhibition. Oncogene. 2007;26(38):5655‐5661. 17334390 10.1038/sj.onc.1210343

